# Status of marine turtle rehabilitation in Queensland

**DOI:** 10.7717/peerj.3132

**Published:** 2017-03-28

**Authors:** Jaylene Flint, Mark Flint, Colin James Limpus, Paul Mills

**Affiliations:** 1 Veterinary-Marine Animal Research, Teaching and Investigation (Vet-MARTI) Unit, School of Veterinary Science, University of Queensland, Gatton, Queensland, Australia; 2 Center for Conservation, The Florida Aquarium, Apollo Beach, FL, United States of America; 3 School of Forest Resources and Conservation, University of Florida, Apollo Beach, FL, United States of America; 4 Department of Environment and Heritage Protection, Threatened Species Unit, Queensland Government, Brisbane, Queensland, Australia

**Keywords:** Sea turtle, Rehabilitation, Stranding, Queensland, Marine turtle

## Abstract

Rehabilitation of marine turtles in Queensland has multifaceted objectives. It treats individual animals, serves to educate the public, and contributes to conservation. We examined the outcome from rehabilitation, time in rehabilitation, and subsequent recapture and restranding rates of stranded marine turtles between 1996 and 2013 to determine if the benefits associated with this practice are cost-effective as a conservation tool. Of 13,854 marine turtles reported as stranded during this 18-year period, 5,022 of these turtles were stranded alive with the remainder verified as dead or of unknown condition. A total of 2,970 (59%) of these live strandings were transported to a rehabilitation facility. Overall, 1,173/2,970 (39%) turtles were released over 18 years, 101 of which were recaptured: 77 reported as restrandings (20 dead, 13 alive subsequently died, 11 alive subsequently euthanized, 33 alive) and 24 recaptured during normal marine turtle population monitoring or fishing activities. Of the turtles admitted to rehabilitation exhibiting signs of disease, 88% of them died, either unassisted or by euthanasia and 66% of turtles admitted for unknown causes of stranding died either unassisted or by euthanasia. All turtles recorded as having a buoyancy disorder with no other presenting problem or disorder recorded, were released alive. In Queensland, rehabilitation costs approximately $1,000 per animal per year admitted to a center, $2,583 per animal per year released, and $123,750 per animal per year for marine turtles which are presumably successfully returned to the functional population. This practice may not be economically viable in its present configuration, but may be more cost effective as a mobile response unit. Further there is certainly benefit giving individual turtles a chance at survival and educating the public in the perils facing marine turtles. As well, rehabilitation can provide insight into the diseases and environmental stressors causing stranding, arming researchers with information to mitigate negative impacts.

## Introduction

The nearshore waters of Queensland, Australia, provide important marine turtle nesting and foraging grounds that support a significant proportion of the South Pacific Ocean loggerhead (*Caretta caretta*) genetic stock and the southern and northern Great Barrier Reef green turtle (*Chelonia mydas*) populations (Limpus & Reimer, 1990; Slater et al., 1998; Dobbs, 2001).

Throughout Australia there are numerous marine turtle rehabilitation centres operating with the aim of contributing to the conservation of marine turtle populations (Feck & Hamann, 2013). In recent years rehabilitation centres have played a dual role: (i) saving individuals which may have otherwise died if they had not received medical attention; and (ii) contributing to environmental education and public awareness (Feck & Hamann, 2013); with the former having a two-fold benefit of keeping individuals alive and conservation of the species.

In Australia, rehabilitation does not have national standardised guidelines. Instead, each facility participating in marine animal care and rehabilitation is limited by their facility’s mission and capacity as well as by recommendations imposed by permitting in each region (for example, local government ordinances and state government requirements). For example, the “Code of Practice—Care of Sick, Injured or Orphaned Animals in Queensland” (Department of Environment and Heritage Protection, 1992) is available for reference in Queensland but it is not a required protocol. Consequently, diagnostic procedures, treatment regimes, and duration in care vary among facilities and when compared to other facilities internationally. This does not mean that welfare and animal care are not considered paramount. Several confounding factors apply in Australia with turtles sent to rehabilitation based on field triage, accessibility of the animal to transport and resource availability to retrieve and receive the animal.

As general public awareness for wildlife conservation has increased, there has been a corresponding increase in the numbers of stranded turtles reported, rehabilitated and subsequently released back into the wild with the intent of enhancing wild populations (Feck & Hamann, 2013). Although the rehabilitation of marine megafauna is driven by concern for the welfare of individual animals, the number of rehabilitated individuals may be too small to have any significant effect on the population or species (Moore et al., 2007; Quakenbush, Beckmen & Brower, 2009; Cardona et al., 2012; Feck & Hamann, 2013; Baker, Edwards & Pike, 2015). Further, animals from numerous species often displayed abnormal behaviour, aberrant dispersal patterns, reduced reproductive success and experienced low survival rates post rehabilitation (Fleming & Gross, 1993; Anderson, Gress & Michael Fry, 1996; Nichols et al., 2000; Bettinger & Bettoli, 2002; Nawojchik et al., 2003; Polovina et al., 2006; Altwegg et al., 2008; Mazzoil et al., 2008; Wells et al., 2009; Wolfaardt et al., 2009; Ebner & Thiem, 2009; Thomas et al., 2010; Bellido et al., 2010; Cardona et al., 2012). As well, a large amount of resources (profit organization offsets, labour, infrastructure and public donations) are used annually to rehabilitate marine turtles in Australia but the benefit this is having on marine turtle populations remains unquantified.

Therefore, given the resources used in rehabilitating marine turtles, assessing the capacity of these species to readapt to the wild, including their ability to survive and reproduce, are essential to guarantee that resources allocated are maximising the number of marine turtles contributing to the functional population (Cardona et al., 2012; Baker, Edwards & Pike, 2015; Flint et al., 2015). Queensland has provided an ideal opportunity for a case study of this issue because of the long running programs of routine population monitoring, stranding response and rehabilitation.

We investigated whether different causes of stranding as well as the length of time an animal spends in care influenced the long term survival of individuals during and post rehabilitation. We summarised and analysed the available data to provide rehabilitation facilities with options to undertake this method of species conservation.

## Methods

### Data

Data used in this study were obtained through StrandNet, the Queensland Government’s Department of Environment and Heritage Protection (EHP) statewide database reporting threatened stranded marine turtles for the entire coast of Queensland and adjacent Commonwealth waters as outlined in Flint et al. (2015). In brief, records were received from members of the public, and employees of EHP, Queensland Parks and Wildlife Services (QPWS), Queensland Department of Agriculture, and Fisheries (DAF), rehabilitation/triage centers (including but not limited to ReefHQ, Cairns Turtle Hospital, James Cook University, Quion Island Turtle Rehabilitation Centre, SeaWorld, Australia Zoo Wildlife Hospital and Underwater World-SEA LIFE Aquarium) and the Great Barrier Reef Marine Park Authority (GBRMPA). Information was collated and stored in this central database. Once reports are entered by first responders the information available is verified by regional and state coordinators for standardization. When required, a second opinion on cause of stranding is sought from experts such as wildlife veterinarians and senior environmental scientists. (For more information, see https://www.ehp.qld.gov.au/wildlife/caring-for-wildlife/strandnet-reports.html).

Additional data were obtained from the EHP Queensland Turtle Conservation Project (QTCP), SeaWorld, Australia Zoo Wildlife Hospital and Underwater World-SEA LIFE Aquarium. The data provided by SeaWorld, Australia Zoo Wildlife Hospital and Underwater World-SEA LIFE Aquarium were used to complete data in StrandNet such as outcomes, causes of death and duration of care.

The QTCP database is the Queensland Government’s EHP statewide database which records tagging and tag recaptures for all marine turtles encountered for the entire coast of Queensland and adjacent Commonwealth waters. Records are received from members of the public, trained volunteers and employees of EHP, QPWS, DAF and GBRMPA. Additional tag recoveries are reported by members of the public. Amalgamating these databases produced the first comprehensive dataset of strandings, causes and captures throughout Queensland for 1996 to 2013.

### Categories used for data analysis

Biometrics were assessed using standard measurements, gonad examination and/or dichotomous key characteristics. Age classes were broken down into three broad categories: small immature, large immature and adult-sized based on curved carapace measurements adapted from Limpus (1992) and Limpus, Couper & Read (1994a) and Limpus, Couper & Read (1994b).

Cause of stranding was identified by examining information compiled from first-responders and trained staff who reviewed reports, photos and codes recorded in StrandNet (Flint et al., 2015). All determinations of the cause of stranding were made within the StrandNet reporting mechanisms and verified outside of this study. The cause identified in StrandNet was then used to group causes for this analysis. Turtles often presented with multiple disorders but are recorded in StrandNet as the suspected primary cause of stranding or most obvious condition. For example, an animal presenting with a disease state causing a buoyancy disorder may only be recorded as “disease”; or an animal admitted floating with a fracture is recorded as fracture because it cannot be determined if it was floating before or only after the time of impact.

### Terms used throughout this study

The term *stranding* is used to incorporate all reported sick, injured, incapacitated or dead marine turtles that either were found washed ashore or encountered at sea. It includes turtles which were entangled in synthetic debris including fishing nets and line, as well as turtles which were rescued (Geraci & Lounsbury, 2005; Biddle & Limpus, 2011; Meager & Limpus, 2012; Flint et al., 2015). For each animal, a single primary cause of stranding was recorded.

*Entanglement* is defined as being entrapped in an anthropogenic object such as fishing line/rope/net.

*Fracture* is used to denote any form of fracture to a turtle that is attributable to anthropogenic causes (e.g., boat strike or blunt force trauma).

*Disease* is classified as turtles which exhibit protracted ill health from a cause consistent with a physiological condition and not otherwise caused by anthropogenic activity (e.g., fracture, entanglement). This is often linked with poor body condition.

*Buoyancy disorder* is used to describe turtles which were observed floating and in which no other presenting problem or disorders were observed.

*Unknown* cause of death/stranding was used when a cause could not be accurately determined. In most cases for cause of death this was due to there being no necropsy performed and no obvious external cause identifiable with a gross examination.

For this study, *survivorship* is defined as a turtle being found in good condition at least once after release from rehabilitation. Determination of condition was made based on coming onto a nesting beach or laparoscopic examination of the gonads or via in-water population surveys and being found to be in good condition at each capture. Rehabilitation was deemed non-successful if the animal was reported stranded again (either dead or alive) within the timeframe of the collected dataset (1996–2013).

*Duration in care* was calculated by subtracting the date of outcome, from the original date of admission/stranding.

*Rodeo* is a technique developed to capture turtles during in-water surveys. This technique is presented pictorially in Limpus (1978) and described in depth in Limpus (1985). Briefly this technique involves the searching for turtles by traversing predefined sampling areas via boats; once observed a jumper dives on the turtle in order to catch and bring it on board the vessel. Turtles were only captured during daylight hours. If more than one turtle was seen at the same time, the first to be sighted was pursued.

### Data analysis

Turtles were included if they stranded along the east Queensland coast within the area of latitude −10.78° (Cape York) to −28.16° (Queensland–New South Wales border) and longitude 142.15 to 155° (Flint et al., 2015). Recaptured turtles were included regardless of where they were encountered (e.g., overseas or in New South Wales) if their original stranding was within the defined Queensland coast.

Animals were matched between the databases using unique identifiers, such as titanium tag numbers. To find subsequent recaptures of the same animal, queries were performed with the capture date that was greater than the first recorded stranding.

Outcomes were analyzed two ways: using the actual calculated time until outcome and then grouping time in care into three groups: admission to 7 days in care (short term stay), 7 to 28 days in care (medium term stay) and greater than 28 days in care (long term stay).

R was used to perform all statistical analysis described above (R Core Team, 2014). Results were presented as descriptive statistics with rigor expressed as a standard 95% Confidence Interval. Confidence intervals were selected as they represent the variance within a dynamic population.

When analyzing time period between release and recapture numbers are expressed as averages, ± a standard deviation.

### Rehabilitation costs

The three main rehabilitation facilities in Queensland were approached and asked to provide their estimated annual costs for marine turtle rehabilitation. These costs are derived based on pool maintenance, food, labor and some medical costs. They do not identify general operating costs that are absorbed by the facility. At best, they should be viewed as an estimate.

The three facilities rehabilitated approximately 68% of turtles admitted to rehabilitation in Queensland. The costs supplied were then extrapolated out to account for all rehabilitated turtles in Queensland, at best these costs should be viewed as a low estimate. Calculations were then made based on numbers of turtles admitted to rehabilitation, numbers of animals released from rehabilitation, numbers of turtles encountered again, and numbers of unsuccessful attempts at rehabilitation.

## Results

### Stranded animals sent to rehabilitation

**Figure 1:**
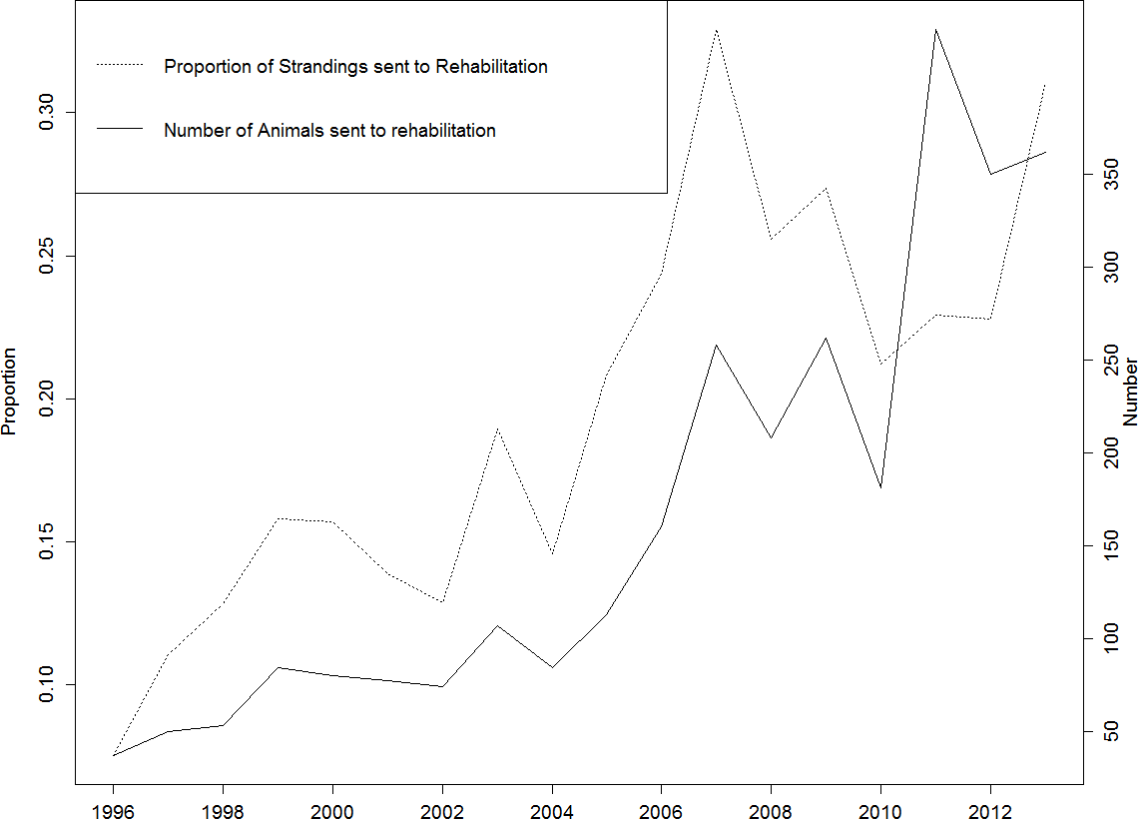
Animals sent to Rehabilitation in Queensland, *n* = 2,970.

Of the 13,854 marine turtle strandings along the Queensland coastline between 1996 and 2013, 5,022 of these animals were stranded alive, of which 2,970 (59.1%, 2970/5022, 95% CI [57.7–60.4]) were admitted to a rehabilitation facility. There was an increase over time in the number (*R*^2^ = 0.70) and the proportion (*R*^2^ = 0.80) of stranded turtles which were sent to rehabilitation facilities in Queensland (Fig. 1).

Two thousand and twenty-one (68%, *n* = 2,021 of the 2,970 rehabilitated turtles) were treated at three institutions: SeaWorld received 596 (20% of turtles sent to rehabilitation, 596/2970, 95% CI [18.6–21.5]), Australia Zoo Wildlife Hospital received 788 (26.5% of turtles sent to rehabilitation, 788/2970, 95% CI [24.9–28.1]), and Underwater World received 637 (21.4% of turtles sent to rehabilitation, 637/2970, 95% CI [20–22.9]).

#### Species of stranded turtles sent to rehabilitation

Green turtles were most often sent to rehabilitation both by number and by proportion (78.2%, 2324/2970, 95% CI [76.7–79.7]). This was followed by hawksbill turtles, *Eretmochelys imbricata* (11.2%, 334/2970, 95% CI [10.1–12.4]) and loggerhead turtles (7.3%, 217/2970, 95% CI [6.4–8.3]) with the other species (flatback turtles, *Natator depressus* (1.2%, 37/2970, 95% CI [0.09–1.7]), olive ridley turtles, *Lepidochelys olivacea* (0.5%, 16/2970, 95% CI [0.3–0.9]), Unknown (1.3%, 41/2970, 95% CI [1–1.8]), black turtle, *Chelonia mydas agassizi* (0.03%, 1/2970, 95% CI [0.006–0.2])) remaining at low levels.

**Figure 2:**
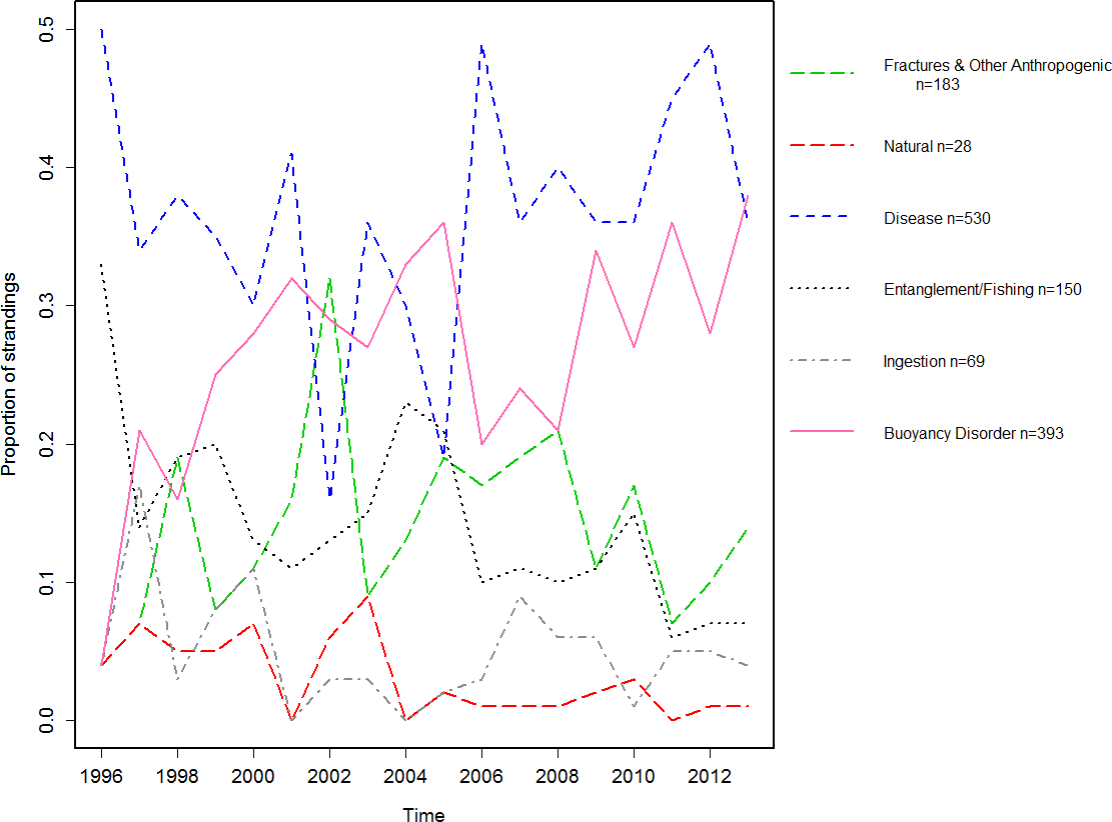
Proportion of animals sent to rehabilitation with an identified cause of stranding.

#### Age class of stranded turtles sent to rehabilitation

Consistently, over the years, the majority of turtles sent to rehabilitation were small immature sized turtles (71%, 2108/2970, 95% CI [69.3–72.6]). The numbers of large immature (15%, 453/2970, 95% CI [14–16.6]) and adult sized (12%, 343/2970, 95% CI [10.4–12.7]) turtles admitted varied each year.

#### Cause of stranding of stranded turtles sent to rehabilitation

The most common cause of stranding for animals sent to rehabilitation was unknown (54%, 1613/2970, 95% CI [52.5–56.1]). The most common identified presenting problems or disorders were disease (18%, 530/2970, 95% CI [16.5–19.2]), buoyancy disorder (13%, 393/2970, 95% CI [12.1–14.5]) and fracture (6%, 167/2970, 95% CI [4.8–6.5]). Figure 2 shows the proportions of animals sent to rehabilitation with identified causes.

#### Age class.

Irrespective of age class, the most common record of stranding for turtles sent to rehabilitation by proportion was from unknown reasons, followed by disease and buoyancy disorder (Table 1).

**Table 1:** Cause of stranding for turtles going to rehabilitation by age-class. Recorded as percentages per age-class.

	Adult sized	Small immatures	Large immatures
Buoyancy disorder	11.95	13.71	13.47
Depredation	2.33	0.33	1.1
Disease	21.57	17.41	18.1
Dredging	0	0.05	0
Entangled ghost fishing	0	0.09	0
Entanglement crabbing	6.41	0.43	0.66
Entanglement fishing	2.04	2.37	1.99
Entanglement rope	0.58	0.38	0
Fractures	8.75	4.65	8.17
Hunting	0.58	0	0.88
Ingestion of foreign material	3.21	1.85	3.97
Land reclamation	0	0	0.44
Nesting beach	0.29	0	0
Netting	0	0.24	0
Other anthropogenic	0.29	0.43	1.32
Unknown	41.11	57.69	47.90
Unknown natural	0.00	0.28	0.44
SCP	0.87	0.09	1.55

**Table 2:** Proportion of animals sent to rehabilitation by species and cause of stranding. Reported as percentages per species.

Cause of stranding	Green turtle (*Chelonia mydas*)	Loggerhead turtle, (*Caretta caretta*)	Hawksbill turtle, (*Eretmochelys imbricata)*	Olive ridley turtle, *(Lepidochelys olivacea)*	Flatback turtle, (*Natator depressus)*	Black turtle *(Chelonia mydas agassizi)*
Buoyancy disorder	13.77	6.45	11.68	18.75	40.54	0
Depredation	0.56	2.3	0	6.25	2.7	0
Disease	18.33	7.83	23.35	25	10.81	0
Dredging	0.04	0	0	0	0	0
Entangled ghost fishing	0.09	0	0	0	0	0
Entanglement crabbing	1.2	2.3	0	0	0	0
Entanglement fishing	2.24	2.3	2.4	0	2.7	0
Entanglement rope	0.39	0	0.9	0	0	0
Fractures	6.2	6.45	2.1	0	2.7	0
Hunting	0.26	0	0	0	0	0
Ingestion of foreign material	2.62	1.38	0.9	6.25	0	100
Land reclamation	0.09	0	0	0	0	0
Nesting beach rescues	0	0.46	0	0	0	0
Netting	0.22	0	0	0	0	0
Other anthropogenic	0.56	0.92	0.3	0	0	0
SCP	0.3	3.69	0.3	0	0	0
Unknown	52.97	64.98	58.08	43.75	35.14	0
Unknown natural	0.17	0.92	0	0	5.41	0

**Figure 3:**
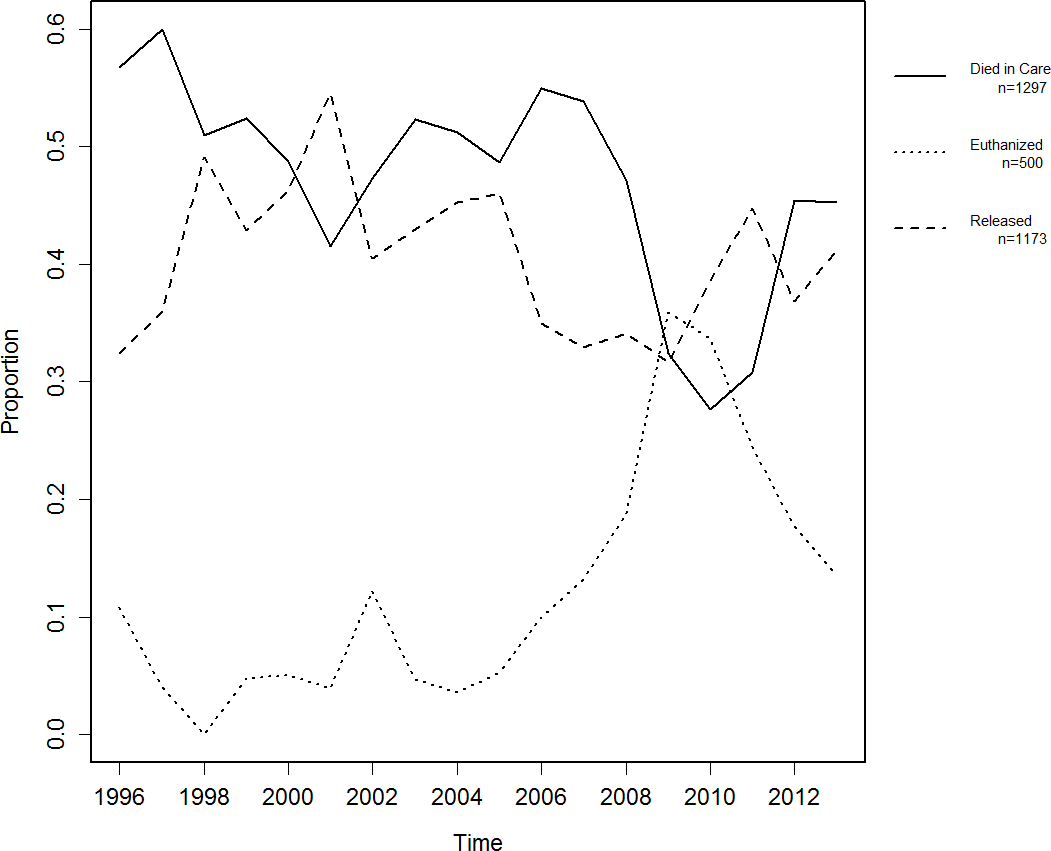
Proportions of animals per outcome.

#### Species.

For all species with the exception of the black and flatback turtles, the most common cause of turtles being sent to rehabilitation was for unknown reasons followed by disease then buoyancy disorders (Table 2). For loggerhead turtles, fractures had the same proportion as buoyancy disorders. For flatback turtles, the most common cause was unknown, followed by buoyancy disorder then disease, and the only black turtle was admitted with ingestion of foreign material.

### Fate of turtles sent to rehabilitation

Between 1996 and 2013, there were changes in the outcomes of turtles sent to rehabilitation (Fig. 3). Overall between 1996 and 2013, the proportion of turtles euthanized increased (*R*^2^ = 0.47), while the proportion of turtles which died while in care decreased (*R*^2^ = 0.38). Between 1996 and 2013, the proportion of turtles which were released was highly variable with a slight decrease (*R*^2^ = 0.07).

Between 2005 and 2009, there was a steady increase in the proportion of turtles euthanized which reversed after this time. During this same period there was an inversely proportional decrease in the number of unassisted deaths in care (Fig. 3). Of the turtles which were sent to rehabilitation, 39% (1173/2970, 95% CI [37.7–41.2]) were released (Table 3).

When combining the proportion of animals which died while in care (assisted and unassisted) and comparing against the number of animals which were released, the proportion of animals which died slightly increased over time (*R*^2^ = 0.07), while the proportion of animals which were released decreased over time (*R*^2^ = 0.07). Both patterns showed a lot of variability.

#### Cause of stranding for euthanized, died unassisted and released rehabilitated turtles

Table 3 shows the outcomes for all turtles sent to rehabilitation (*n* = 2,970). Overall, 500 rehabilitated turtles were euthanized. The cause of stranding for the highest number euthanized were unknown (48.7%, 239/500, 95% CI [43.4–52.2]), followed by disease (31.4%, 157/500, 95% CI [27.5–35.6]) and fractures (12.4%, 62/500, 95% CI [9.8–15.6]).

Between 1996 and 2013, 1,297 stranded and rehabilitated turtles died unassisted while in care. The highest number of these turtles died for unknown reasons (63%, 818/1297, 95% CI [60.4–65.6]), followed by disease (23.8%, 309/1297, 95% CI [21.6–26.2]) and fractures (4.8%, 63/1297, 95% CI [3.8–6.1]).

Between 1996 and 2013, 1,173 stranded and rehabilitated turtles were released from rehabilitation alive. The number of turtles which were released for each stranding cause was variable, including unknown reasons (47.4%, 556/1173, 95% CI [44.5–50.3]), buoyancy disorder (33.5%, 393/1173, 95% CI [30.8–36.2]), and disease (5.4%, 64/1173, 95% CI [4.3–6.9]).

#### Time in care for euthanized, died unassisted and released rehabilitated turtles

Two thousand four hundred and ninety four stranded and rehabilitated turtles had duration of care recorded (84% of all animals admitted to rehabilitation), 1,139 of which died unassisted in care (45.7%), 480 were euthanized (19.2%) and 875 were released (35.1%). When analyzing duration of care across all outcomes this was compared as a combined total of turtles sent to rehabilitation.

Table S1 shows the grouped duration in care before outcome, with 6.6% (165/2494, 95% CI [5.7–7.7]) of all turtles were released within the first 7 days, 4.2% (106/2494, 95% CI [3.5–5.1]) of all turtles were released between days 7 and 28, and 24.2% (604/2494, 95% CI [22.6–26.9]) were released after 28 days. Since 1999, the average days in care before release decreased from 392 to 84 days, but the minimum days in care before release remained low at an average of 0.74 of a day (Table S1).

Table S1 shows the changes over the years in duration in care over the years; the average number of days in care before being euthanized varied over the years, with an overall decrease. 11.9% (298/2494, 95% CI [10.7–13.3]) of all turtles were euthanized in the first 7 days, 3.9% (98/2494, 95% CI [3.2–54.8] of all turtles were euthanized between 7 and 28 days, and 3.3% (84/2494, 95% CI [2.7–4.1]) of all turtles were euthanized after 28 days (Table S1).

Between 1996 and 2013, 25.4% (634/2494, 95% CI [23.7–27.2]) of all turtles died without assistance within the first 7 days, 11.3% (283/2494, 95% CI [10.1–12.6]) died between days 7 and 28, 8.9% (222/2494, 95% CI [7.8–10.1]) died after the first 28 days (Table S2). In 1997 there was a spike in the average number of days in care before unassisted death after which the average days in care before death occurred decreased from 41–15 days.

### Recaptures of turtles sent to rehabilitation

Between 1996 and 2013, of the 1,173 turtles released from rehabilitation, 101 turtles were recaptured (Table 4). This represented 8.6% (101/1173, 95% CI [7.1–10.4]) of the turtles released from rehabilitation.

**Table 3:** Cause of stranding distributed by outcome of rehabilitation. The numbers by outcome, animals and cause are reported as percentages.

Cause of stranding	Died in care				Euthanized				Released			
	*n*	% by outcome	% of all animals	% by cause	*n*	% by outcome	% of all animals	% by cause	*n*	% by outcome	% of all animals	% by cause
Buoyancy disorder	0	0	0	0	0	0	0	0	393	33.50	13.23	100
Depredation	9	0.6	0.30	45	2	0.40	0.07	10	9	0.77	0.30	45
Disease	309	23.8	10.40	58.30	157	31.40	5.29	29.62	64	5.46	2.15	12.08
Dredging	1	0.08	0.03	100	0	0	0	0	0	0	0	0
Entangled ghost fishing	0	0	0	0	1	0.20	0.03	25	3	0.26	0.10	75
Entanglement crabbing	2	0.15	0.07	5.41	2	0.40	0.07	5.41	33	2.81	1.11	89.19
Entanglement fishing	17	1.31	0.57	24.64	12	2.40	0.40	17.39	40	3.41	1.35	57.97
Entanglement rope	5	0.39	0.17	38.46	2	0.40	0.07	15.38	6	0.51	0.20	46.15
Fractures	63	4.8	2.12	37.72	62	12.40	2.09	37.13	42	3.58	1.41	25.15
Hunting	0	0		0	0	0	0	0	6	0.51	0.20	100
Ingestion of foreign material	49	3.78	1.65	71.01	19	3.80	0.64	27.54	1	0.09	0.03	1.45
Land reclamation	0	0	0	0	0	0	0	0	2	0.17	0.07	100
Nesting beach rescues	3	0.23	0.10	60	0	0	0	0	2	0.17	0.07	40
Netting	0	0	0	0	1	0.20	0.03	20.00	4	0.34	0.13	80
Other anthropogenic	13	0	0.44	81.25	2	0.40	0.07	12.50	1	0.09	0.03	6.25
SCP	5	0.39	0.17	31.25	1	0.20	0.03	6.25	10	0.85	0.34	62.50
Unknown	818	63.07	27.54	50.71	239	47.80	8.05	14.82	556	47.4	18.72	34.47
Unknown natural	3	0.23	0.10	75	0	0	0	0	1	0.09	0.03	25
**Grand total**	**1297**	**100**	**43.67**	**NA**	**500**	**100**	**16.84**	**NA**	**1173**	**100**	**39.49%**	**NA**

**Table 4:** Original reason for stranding, with subsequent recapture reason and fate.

Original cause of stranding	Subsequent recapture method	Alive	Alive and subsequently died unassisted	Dead	Euthanized	Grand total
**Buoyancy disorder**		**22**	**4**	**4**	**3**	**33**
	Fracture	1	0	0	0	1
	Disease	3	1	0	1	5
	Entanglement fishing	2	0	0	0	1
	Buoyancy disorder	3	0	0	0	3
	Unknown	9	3	4	2	18
	Rodeo	4	0	0	0	4
**Depredation**		**1**	**1**	**0**	**0**	**2**
	Unknown	1	1	0	0	2
**Disease**		**5**	**0**	**0**	**2**	**7**
	Disease	1	0	0	1	2
	Buoyancy disorder	1	0	0	0	1
	Unknown	2	0	0	1	3
	Rodeo	1	0	0	0	1
**Entanglement crabbing**		**1**	**0**	**0**	**0**	**1**
	Nesting	1	0	0	0	1
**Entanglement fishing**		**2**	**0**	**1**	**0**	**3**
	Unknown	2	0	1	0	3
**Entanglement rope**		**1**	**0**	**0**	**0**	**1**
	Rodeo	1	0	0	0	1
**Fractures**		**3**	**0**	**0**	**0**	**3**
	Unknown	1	0	0	0	1
	Rodeo	1	0	0	0	1
	Nesting	1	0	0	0	1
**SCP**		**5**	**0**	**0**	**0**	**5**
	Entanglement fishing	1	0	0	0	1
	SCP	4	0	0	0	4
**Unknown**		**17**	**8**	**15**	**6**	**46**
	Disease	0	1	0	4	5
	Buoyancy disorder	2	0	0	0	2
	Unknown	7	7	15	2	31
	Rodeo	8	0	0	0	8
**Grand total**		**57**	**13**	**20**	**11**	**101**

Of the turtles released from rehabilitation and subsequently recaptured, 76.2% (77/101, 95% CI [67.1–83.4]) were recorded as restranded and 17.8% (18/101, 95% CI [11.6–26.4]) of them were recorded during normal population studies. Of the remaining six turtles, 5.9% (6/101, 95% CI [2.7–12.4]) were recaptured during fishing activities.

Twenty of the turtles that subsequently restranded were dead (1–2,820 days after release, average 485 days ± 725), 11 were alive and subsequently euthanized (1–2,534 days after release, average 446 days ± 720), 13 were alive and subsequently died unassisted (1–1,619 days after release, average 356 days ± 517) and 33 were alive and re-admitted for rehabilitation (1–1,130 days after release, average 225 ± 284).

#### Recapture/restranding cause compared to original stranding cause

The most common original cause of stranding for turtles that were recaptured alive was buoyancy disorder (39.6%, 22/57, 95% CI [27.1–51.2]); followed by unknown original cause of stranding (29.8%, 17/57, 95% CI [19.5–42.6]); all other original causes contributed a total of 31%. For the nine identified categories of restranding; seven of these categories (depredation, disease, entanglement in fishing gear, entanglement in rope, buoyancy disorder, shark control program (SCP), and unknown causes) showed there was more than a 50% chance the turtle would restrand for the same reason as it originally stranded (Table 4).

#### Cost of rehabilitation

Collectively, SeaWorld, Australia Zoo Wildlife Hospital and Underwater World-SEA LIFE Aquarium reported spending a total average of $AUD112,000 per annum on marine turtle rehabilitation to treat approximately two thirds (68%) of all marine turtles received for rehabilitation. Extrapolating from the annual amount spent at SeaWorld, Australia Zoo Wildlife Hospital and Underwater World-SEA LIFE Aquarium and assuming that other facilities have a similar expenditure, this approximates $AUD165,000 being spent per annum in Queensland across the participating rehabilitation facilities on marine turtle rehabilitation.

Over the 18 years of this study, 2,970 turtles were admitted to all rehabilitation facilities in Queensland, equating to an average of 165 turtles admitted per annum, costing approximately $AUD1,000 per animal admitted if you average total money spent on marine turtle rehabilitation per animal admitted. Over the 18 years of this study, 1,173 of these turtles were released, equaling approximately 65 turtles per year at an estimated cost of $AUD2,583 per animal released from rehabilitation if you average total money spent on marine turtle rehabilitation per animal released. Over the 18 years of this study, 101 of these released turtles were recaptured, equaling an average of 5.6 turtles per year and costing on average $AUD29,464 to rehabilitate each animal that is caught again. Of all of these animals admitted across all rehabilitation facilities for marine turtles, rehabilitated, released and recaptured, only 24 turtles were recaptured as functioning healthy members of the wild population, equaling approximately 1.3 turtles per year at a cost of $AUD123, 750 to return a single animal to the functional population.

When analyzing the costs for animals which were not successful during rehabilitation it is estimated that approximately $AUD1650 is spent per turtle which is either euthanized or dies while in care.

## Discussion

This investigation found that different causes of stranding influenced the survival for individuals, in terms of length of time in care, and survival of rehabilitation and post rehabilitation success. This provides rehabilitation centres with important information about resource outlay, particularly if success rates are poor (approximately 8.6%).

When analysing stranding data for Queensland between 1996 and 2013, Flint et al. (2015) found several significant trends in stranding numbers including: (i) an increase in the number of turtles reported stranded in Queensland during the study period (*R*^2^ = 0.6377); (ii) a species (loggerhead and green turtles (77.4%)) prevalence; (iii) a seasonal effect on different age classes, with most overall strandings occurring between August and November (47%); and (iv) stranding hotspots (Moreton Bay, Hervey Bay, Rockhampton region and Cleveland Bays) persisting throughout the study timeframe. These hotspots correspond to major freshwater discharge points as well as highly developed/populated areas.

Green turtles were the most frequent species and immature turtles were the most frequent age class sent to rehabilitation both by numbers and proportion, most likely because green turtles represent the largest proportion of the Queensland marine turtle populations and small immature turtles are the largest cohort of this population (Chaloupka & Limpus, 2001; Chaloupka, 2002a). Further, small immature turtles are likely to be the most susceptible cohort as a result of having a naïve immune system (Flint et al., 2010a; Flint et al., 2010b) to numerous potential stressors and being obligate residents of nearshore habitats that may be subject to a range of environmental stressors (Limpus, Couper & Read, 1994b; Flint et al., 2010a; Flint et al., 2010b). The larger number of small immature turtles being found is further compounded with them being nearshore residents, which has been shown to increase the likelihood of recorded strandings (Peltier et al., 2012).

Increased morbidity and mortality is often associated with the perils of anthropogenic interactions. Green turtle stranding increased at a rate of 9.9% per annum (pa) over the study period. However, during a similar timeframe, the southern Great Barrier Reef green turtle population increased at a rate of approximately 10.6% pa (Chaloupka & Limpus, 2001). As well, the southern Great Barrier Reef foraging loggerhead turtle populations declined over this same period at approximately 3% pa (Chaloupka & Limpus, 2001) and stranding rates decreased at a rate of 3.5% pa. Stranding numbers in Queensland may be a normal function of the population and a proxy for the overall population change when it is in decline.

For stranded turtles which were recaptured, the primary known presenting problem or disorder were disease, buoyancy disorders and fractures (trauma) (Fig. 2). As the former two signs may both represent multiple conditions, successful treatment within rehabilitation centers requires the determination of the cause of stranding diagnosis/underlying health problem. Potentially as a result of this limitation, the most common causes of restrandings were turtles that originally presented with one of these two conditions. Conversely, rehabilitation centers traditionally have a good success rate identifying and treating trauma for complete release with very few of these turtles restranding. Further, our study suggested there are certain times of the year when it can be expected certain conditions are going to present. Disease and buoyancy disorders were highest at the end of winter, likely when resources were stressed and immune systems were under duress. Trauma was most prevalent during the summer months when recreational boating may be at its greatest as seen in other popular urbanized embayments (Widmer & Underwood, 2004). Despite the introduction of “go-slow zones” for motorized crafts there has been an overall increase in the proportion of turtles admitted to rehabilitation with fractures. This may not just result from the obvious threat of recreational or commercial boating but could include: (i) more turtles sustaining non-life threatening injuries enabling them to survive and be taken to rehabilitation; (ii) an increase in public awareness increasing the number of animals being taken to rehabilitation; or (iii) the population of turtles is increasing (Chaloupka & Limpus, 2001), increasing the likelihood of a negative encounter with a recreational vessel. In all cases, trauma may be reduced by improved restrictions in certain zones on a seasonal basis; such as has been successfully employed to protect the Florida Manatee (*Trichechus manatus latirostris*) in high use areas (Calleson & Frohlich, 2007; Calleson, 2014).

For a short period between 2005 and 2009, euthanasia rates increased across rehabilitation centers in Queensland (Fig. 3). As there were no recorded epidemics during this time, reasons for this trend remain unclear. There was no significant shift in expertise during this time and the majority of new rehabilitation centers opened after the 2010 major floods (Meager & Limpus, 2012). A potential for this peak in 2009 is an oil spill which occurred in the northern Moreton Bay Area (SEQ Catchments, 2011) but this does not account for the 4-year period prior to this catastrophe. Funding to individual rehabilitation centers or recommended treatment regimens may have influenced this peak.

During rehabilitation, over 25% of turtles die unassisted during the first week of treatment (Table S2), suggesting progression of cause of stranding is too advanced or the disease syndrome is too complex for treatment and successful reintroduction to the population. This phenomenon must be addressed to ensure diagnostic regimes, animal welfare and limited resources are being optimally used. Anthropogenic causes (not including fractures) were least successfully treated. A likely reason is the degree of intervention required when compared with diseases that can be systemically treated with appropriate palliative care (Table 3).

Some of the results found in this study differ from that of Bosagna (2012), who found that only 7.14% of turtles admitted to rehabilitation died unassisted in the first 15 days of treatment. Possible reasons for this are that Bosagna (2012) only analyzed 2 years of data from three rehabilitation facilities. The results for turtles that were released and turtles which were euthanized were similar when the initial time periods are compared (<15 days (Bosagna, 2012), and >7 days (this study)).

Similar rehabilitation results were obtained by Baker, Edwards & Pike (2015) who looked at the outcome of turtles from rehabilitation in Florida. They found that 36.8% of turtles admitted to rehabilitation were released back into the wild, while 55.3% died while in rehabilitation. This suggests treatment regimens and approaches and stressors between the USA and Australia may be comparable.

It is difficult to assess the true success of rehabilitation without following each individual. There have been few studies investigating the ability of turtles to readapt to the wild after rehabilitation (Tomás et al., 2001; Cardona et al., 2012; Feck & Hamann, 2013). The two most appropriate methods for assessing post-rehabilitation survivorship are satellite-tracking individuals or tagging individuals and monitoring for their restranding or recapture with time. Queensland has provided an ideal opportunity for a case study of this issue because of the long running programs of both stranding and routine population monitoring. Shimada et al. (2016) analyzed satellite tracking data of turtles which had been displaced from their original capture site (inferred home area). Of the 59 displaced turtles, 52 returned to their home areas. All 52 non-displaced turtles remained within their home areas. This indicates that turtles which are removed from their home area are likely to return to that location. It follows that if turtles were exposed to threats at the original location, once they return from rehabilitation they will possibly be exposed to those same threats again and hence potentially succumb and restrand. This study showed the original cause of stranding is closely linked to the cause of restranding suggesting either incomplete treatment during rehabilitation (e.g., not eliminating the disease during treatment) or re-exposure or behavioral predisposition in certain turtles to recreate the hazard (e.g., SCP or fishing line entanglement).

The number of turtles reported stranded during this study represent approximately >0.001% ((9641 turtles/18 years)/641262) of the suspected benthic southern Great Barrier Reef population (Chaloupka, 2002b). Despite the successes and challenges of rehabilitation, a large number of turtles were released over 18 years, from which 9% were recaptured either as restranded or healthy and part of normal survey activities giving an insight in to the long term outcome of human intervention as a population conservation tool. To put this in context, functional population recapture rates range from 8%–84.3% depending on age class (Chaloupka & Limpus, 2002; Chaloupka & Limpus, 2005; Bell, Schwarzkopf & Manicom, 2012). While this may indicate a large proportion of released turtles are subsequently dying or otherwise being removed from the population, this may not necessarily be the case because some turtles may be released back into an area that is not within regularly sampled regions of those serially surveyed.

Based on statewide recapture data and the cost of rehabilitation at the three main rehabilitation centres in Queensland (Australia Zoo Wildlife Hospital, SeaWorld and Underwater World-SEA LIFE Aquarium), only 1.3 turtles are successfully returned to the functional population each year at a cost of $AUS123,750 an animal. Even though these costs should only be viewed as indicative estimates as opposed to a true calculation per known animal released, the pattern indicates this may not be economical and these high costs and low numbers are likely not contributing to conserving a population (Moore et al., 2007; Quakenbush, Beckmen & Brower, 2009; Cardona et al., 2012; Feck & Hamann, 2013; Baker, Edwards & Pike, 2015). However, rehabilitation may still be benefit the populations through public education, increased awareness and advances in our understanding and treatment of the diseases and biology of marine turtles through this means of conservation. With the high costs associated with unsuccessful cases admitted through rehab, there may be more productive way some of this money could be used to educate the public so that more marine turtles in the wild can benefit (such as boater education, beach protection/monitoring, anti-litter campaigns, fisher education). Similarly, scientist education and development of better protocols and understanding of the underlying processes causing stranding may benefit from funding.

There is no question rehabilitation plays an important role in the care of individual turtles by reducing suffering and treatment of certain conditions. Part of the rehabilitation process can include euthanasia as a treatment option to prevent individual suffering and can add value to research through appropriate post-mortem investigations. Rehabilitation also provides a valuable vehicle for public education and conservation messages which, in turn, increase public awareness and hopefully reduce anthropogenic causes of stranding (Cardona et al., 2012; Feck & Hamann, 2013; Baker, Edwards & Pike, 2015). This rationale adheres to the One Health paradigm, whereby educating people to take more responsibility for their actions can reduce their impacts (by changing perception of how they treat the ocean) that can have a direct impact on the environmental health and resultant animal health (Schwabe, 1969; Flint, 2013). However, with respect to augmenting the population of marine turtles, rehabilitation may not contribute to survivorship. This is influenced by the size and robustness of the local turtle population, the factors affecting stranding and the conservation status of the local population.

Given the prevalence of: (i) certain cohorts; (ii) seasonality; (iii) certain causes of stranding; and (iv) higher numbers of strandings at four locations along the Queensland coastline (hotspots), it may be more appropriate to direct rehabilitation efforts to events of higher demand. For example, this could mean creating MASH (Mobile Army Surgical Hospital)-like response centers that target their care to immature turtles that present at the end of the winter period (August–September)—to treat/evaluate boat strike and unknown causes, within the recognized stranding ‘hotspots’. Focused triage and treatment may represent a significant cost saving to rehabilitation centers throughout Australia. As 22% of animals which are admitted to rehabilitation reach an outcome within 7 days, the creation of such response centers, will allow turtles with obvious disorders which only need short term immediate care to be treated and released, freeing up resources and space in rehabilitation centers for turtles which require more in-depth/long-term care.

Despite the costs involved, rehabilitation continues to be a tool for conservation because it provides a platform to educate members of the public about threats to marine turtle survival (Addison & Nelson, 2000; Feck & Hamann, 2013). It has been shown that when people visit zoos or aquaria that have a prominent conservation message, the visitors’ mindsets can be changed towards being more pro-conservation (Adelman, Falk & James, 2000; Falk et al., 2007; Wyles et al., 2013). Rehabilitation also provides insight into the diseases causing stranding through ancillary investigations (Flint et al., 2010b). Information from post-mortem investigations such as necropsy can help first-responders to gather insight into what disease and parasitic prevalence may be present during normal times to create a baseline of “background” pathologies for a region. This in turn may aid in determining when a syndrome becomes an outbreak (or unusual mortality event) and allows future first-responders to be better prepared.

##  Supplemental Information

10.7717/peerj.3132/supp-1 Table S1 Duration of care before outcome. Short term care 0–7 days, Medium term care 7–28 days, Long term care >28 days

10.7717/peerj.3132/supp-2 Table S2 Duration in care before outcome for each reported cause of stranding

10.7717/peerj.3132/supp-3 Data S1 Raw data used for this study

## Additional Information and Declarations

### Data Availability

The following information was supplied regarding data availability: The raw data has been supplied as a Supplementary File.

## References

[ref-1] Addison DS, Nelson KA (2000). Recapture of a tagged, captive reared juvenile loggerhead turtle—an example of habituation?. Marine Turtle Newsletter.

[ref-2] Adelman LM, Falk JH, James S (2000). Impact of national aquarium in Baltimore on visitors’ conservation attitudes, behavior, and knowledge. Curator.

[ref-3] Altwegg R, Crawford RJM, Underhill LG, Williams AJ (2008). Long-term survival of de-oiled Cape gannets *Morus capensis* after the Castillo de Bellver oil spill of 1983. Biological Conservation.

[ref-4] Anderson DW, Gress F, Michael Fry D (1996). Survival and dispersal of oiled brown pelicans after rehabilitation and release. Marine Pollution Bulletin.

[ref-5] Baker L, Edwards W, Pike D (2015). Sea turtle rehabilitation success increases with body size and differs among species. Endangered Species Research.

[ref-6] Bell IP, Schwarzkopf L, Manicom C (2012). High survivorship of an annually decreasing aggregation of hawksbill turtles, *Eretmochelys imbricata*, found foraging in the northern Great Barrier Reef. Aquatic Conservation: Marine and Freshwater Ecosystems.

[ref-7] Bellido JJ, Báez JC, Castillo JJ, Martín JJ, Mons JL, Real R (2010). Unusual behaviour of an immature loggerhead turtle released in the Alboran Sea. Animal Biodiversity and Conservation Biology.

[ref-8] Bettinger JM, Bettoli PW (2002). Fate, dispersal, and persistence of recently stocked and resident rainbow trout in a Tennessee tailwater. North American Journal of Fisheries Management.

[ref-9] Biddle T, Limpus CJ (2011). Marine wildlife stranding and mortality database annual reports 2005–2010. Marine turtles.

[ref-10] Bosagna RG (2012). The rehabiliation and release of marine fauna in south east Queensland: green sea turtles (*Chelonia mydas*) as a case study.

[ref-11] Calleson C (2014). Issues and opportunities associated with using manatee mortality data to evaluate the effectiveness of manatee protection efforts in Florida. Endangered Species Research.

[ref-12] Calleson C, Frohlich R (2007). Slower boat speeds reduce risks to manatees. Endangered Species Research.

[ref-13] Cardona L, Fernández G, Revelles M, Aguilar A (2012). Readaptation to the wild of rehabilitated loggerhead sea turtles (*Caretta caretta*) assessed by satellite telemetry. Aquatic Conservation: Marine and Freshwater Ecosystems.

[ref-14] Chaloupka M (2002a). Stochastic simulation modelling of southern Great Barrier Reef green turtle population dynamics. Ecological Modelling.

[ref-15] Chaloupka M (2002b). Phase 1—assessment of suitability of Queensland Parks & Wildlife Service Sea Turtle Data for use in models of the population dynamics of the Southern Great Barrier Reef Turtle Stock. Great Barrier Reef Marine Park Authority Research Publication.

[ref-16] Chaloupka M, Limpus CJ (2001). Trends in the abundance of sea turtles resident in southern Great Barrier Reef waters. Biological Conservation.

[ref-17] Chaloupka M, Limpus CJ (2002). Survival probability estimates for the endangered loggerhead sea turtle resident in southern Great Barrier Reef waters. Marine Biology.

[ref-18] Chaloupka M, Limpus CJ (2005). Estimates of sex- and age-class-specific survival probabilities for a southern Great Barrier Reef green sea turtle population. Marine Biology.

[ref-19] Department of Environment and Heritage Protection (1992). Code of Practice—Care of sick, injured or orphaned protected animals in Queensland. Nature Conservation Act 1992.

[ref-20] Dobbs K (2001). Marine Turtles in the Great Barrier Reef World Heritage Area: a compendium of information and basis for the development of policies and strategies for the conservation of marine turtles.

[ref-21] Ebner BC, Thiem JD (2009). Monitoring by telemetry reveals differences in movement and survival following hatchery or wild rearing of an endangered fish. Marine and Freshwater Research.

[ref-22] Falk JH, Reinhard EM, Vernon CL, Bronnenkant K, Heimlich JE, Deans NL (2007). Why zoos & aquariums matter: assessing the impact of a visit to a zoo or aquarium. Association of Zoos Aquariums.

[ref-23] Feck AD, Hamann M (2013). Effect of sea turtle rehabilitation centres in Queensland, Australia, on people’s perceptions of conservation. Endangered Species Research.

[ref-24] Fleming IA, Gross MR (1993). Breeding success of hatchery and wild coho salmon (*Oncorhynchus kisutch*) in competition. Ecological Applications.

[ref-25] Flint M, Wyneken J, Lohmann KJ, Musick JA (2013). Free-ranging sea turtle health. The biology of sea turtles.

[ref-26] Flint J, Flint M, Limpus CJ, Mills PC (2015). Trends in marine turtle strandings along the East Queensland, Australia Coast, between 1996 and 2013. Journal of Marine Biology.

[ref-27] Flint M, Morton JM, Limpus CJ, Patterson-Kane JC, Murray PJ, Mills PC (2010a). Development and application of biochemical and haematological reference intervals to identify unhealthy green sea turtles (*Chelonia mydas*). The Veterinary Journal.

[ref-28] Flint M, Patterson-Kane JC, Limpus CJ, Mills PC (2010b). Health surveillance of stranded green turtles in Southern Queensland, Australia (2006–2009): an epidemiological analysis of causes of disease and mortality. EcoHealth.

[ref-29] Geraci JR, Lounsbury VJ (2005). Marine mammals ashore: a field guide for strandings.

[ref-30] Limpus CJ (1978). The reef: uncertain land of plenty. Exploration north, a natural history of Queensland.

[ref-31] Limpus CJ (1985). A study of the loggerhead sea turtle, *Caretta caretta*, in Eastern Australia.

[ref-32] Limpus CJ (1992). The hawksbill turtle, *Eretmochelys imbricata*, in Queensland: population structure within a southern Great Barrier Reef feeding ground. Wildlife Research.

[ref-33] Limpus CJ, Couper PJ, Read MA (1994a). The green turtle, *Chelonia mydas*, in Queensland: population structure in a warm temperate feeding area. Memoirs of the Queensland Museum.

[ref-34] Limpus CJ, Couper PJ, Read MA (1994b). The loggerhead turtle, *Caretta caretta*, in Queensland: population structure in a warm temperate feeding area. Memoirs of the Queensland Museum.

[ref-35] Limpus CJ, Reimer D (1990). The loggerhead turtle, *Caretta caretta* in Queensland: a population in decline.

[ref-36] Mazzoil MS, McCulloch SD, Youngbluth MJ, Kilpatrick DS, Murdoch ME, Mase-Guthrie B, Odell DK, Bossart GD (2008). Radio-tracking and survivorship of two rehabilitated bottlenose dolphins (*Tursiops truncatus*) in the Indian River Lagoon, Florida. Aquatic Mammals.

[ref-37] Meager JJ, Limpus CJ (2012). Marine wildlife stranding and mortality database annual report 2011 III. Marine Turtles.

[ref-38] Moore M, Early G, Touhey K, Barco SG, Gulland F, Wells RS (2007). Rehabilitation and release of marine mammals in the United States: risks and benefits. Marine Mammal Science.

[ref-39] Nawojchik R, Aubin DJS, Johnson A, Aquarium M, Boulevard C, Aquarium M, Aquarium E, Aquarium NE, Wharf C, Nawojchik R, Aubin DJS, Johnson A (2003). Movements and dive behavior of two stranded, rehabilitated long-finned pilot whales (*Globicephala melas*) in the Northwest Atlantic. Marine Mammal Science.

[ref-40] Nichols WJ, Resendiz A, Seminoff JA, Resendiz B (2000). Transpacific migration of a loggerhead turtle monitored by satellite telemetry. Bulletin of Marine Science.

[ref-41] Peltier H, Dabin W, Daniel P, Van Canneyt O, Dorémus G, Huon M, Ridoux V (2012). The significance of stranding data as indicators of cetacean populations at sea: modelling the drift of cetacean carcasses. Ecological Indicators.

[ref-42] Polovina J, Uchida I, Balazs GH, Howell EA, Parker D, Dutton PH (2006). The Kuroshio Extension Bifurcation Region: a pelagic hotspot for juvenile loggerhead sea turtles. Deep Sea Research Part II: Topical Studies in Oceanography.

[ref-43] Quakenbush L, Beckmen K, Brower CDN (2009). Rehabilitation and release of marine mammals in the United States: concerns from Alaska. Marine Mammal Science.

[ref-44] R Core Team (2014). R: a language and environment for statistical computing.

[ref-45] Schwabe CW (1969). Veterinary medicine and human health.

[ref-46] SEQ Catchments (2011). The Moreton Bay oil spill, environmental restoration program.

[ref-47] Shimada T, Limpus CJ, Jones R, Hazel J, Groom R, Hamann M (2016). Sea turtles return home after intentional displacement from coastal foraging areas. Marine Biology.

[ref-48] Slater J, Limpus CJ, Robins J, Pantus F, Chaloupka M (1998). Risk assessment of sea turtle capture in the Queensland east coast otter trawl fishery.

[ref-49] Thomas K, Harvey JT, Goldstein T, Barakos J, Gulland F (2010). Movement, dive behavior, and survival of California sea lions (*Zalophus californianus*) post treatment for domoic acid toxicosis. Marine Mammal Science.

[ref-50] Tomás J, Dominici A, Nannarelli S, Forni L, Badillo FJ, Raga JA (2001). From hook to hook: the odyssey of a loggerhead sea turtle in the Mediterranean. Marine Turtle Newsletter.

[ref-51] Wells RS, Manire CA, Byrd L, Smith DR, Gannon JG, Fauquier D, Mullin KD (2009). Movements and dive patterns of a rehabilitated Risso’s dolphin, *Grampus griseus*, in the Gulf of Mexico and Atlantic Ocean. Marine Mammal Science.

[ref-52] Widmer W, Underwood A (2004). Factors affecting traffic and anchoring patterns of recreational boats in Sydney Harbour, Australia. Landscape and Urban Planning.

[ref-53] Wolfaardt AC, Williams AJ, Underhill LG, Crawford RJM, Whittington PA (2009). Review of the rescue, rehabilitation and restoration of oiled seabirds in South Africa, especially African penguins *Spheniscus demersus* and Cape gannets *Morus capensis*, 1983–2005. African Journal of Marine Science.

[ref-54] Wyles KJ, Pahl S, White M, Morris S, Cracknell D, Thompson RC (2013). Towards a marine mindset: visiting an aquarium can improve attitudes and intentions regarding marine sustainability. Visitor Studies.

