# Supplementary material for: Status of marine turtle rehabilitation in Queensland
Source: PeerJ. 2017 Mar 28;5:e3132. doi: 10.7717/peerj.3132 (PMC5372840; doi:10.7717/peerj.3132)
Supplement: Table S1 [file peerj-05-3132-s001.docx]

|  | **n** | **Min** | **Max** | **Average** | **Numbers in short term** | **Numbers in medium term care** | **Numbers in long term care** |
| --- | --- | --- | --- | --- | --- | --- | --- |
| **1996** |  |  |  |  |  |  |  |
| **Released** | 8 | 5 | 271 | 94.75 | 1 | 1 | 6 |
| **Died in Care** | 17 | 0 | 378 | 40.82 | 9 | 5 | 3 |
| **Euthanized** | 4 | 7 | 136 | 49.25 | 0 | 2 | 2 |
| **1997** |  |  |  |  |  |  |  |
| **Released** | 13 | 0 | 715 | 143.85 | 3 | 0 | 10 |
| **Died in Care** | 16 | 0 | 2720 | 203.56 | 7 | 3 | 6 |
| **Euthanized** | 0 | NA | NA | NA |  |  |  |
| **1998** |  |  |  |  |  |  |  |
| **Released** | 15 | 0 | 1076 | 189.73 | 2 | 3 | 10 |
| **Died in Care** | 9 | 0 | 365 | 55.44 | 6 | 0 | 3 |
| **Euthanized** | 0 | NA | NA | NA |  |  |  |
| **1999** |  |  |  |  |  |  |  |
| **Released** | 19 | 0 | 4919 | 392.26 | 4 | 1 | 14 |
| **Died in Care** | 29 | 0 | 243 | 29.79 | 12 | 6 | 11 |
| **Euthanized** | 4 | 6 | 1096 | 449.75 | 1 | 1 | 2 |
| **2000** |  |  |  |  |  |  |  |
| **Released** | 21 | 0 | 3653 | 293.19 | 6 | 4 | 11 |
| **Died in Care** | 39 | 0 | 1361 | 68.41 | 10 | 11 | 8 |
| **Euthanized** | 3 | 4 | 28 | 13.67 | 1 | 1 | 1 |
| **2001** |  |  |  |  |  |  |  |
| **Released** | 33 | 0 | 3798 | 251.94 | 6 | 3 | 24 |
| **Died in Care** | 23 | 0 | 1463 | 118.13 | 12 | 3 | 8 |
| **Euthanized** | 3 | 0 | 23 | 14.33 | 1 | 2 | 0 |
| **2002** |  |  |  |  |  |  |  |
| **Released** | 24 | 1 | 312 | 80.25 | 3 | 2 | 19 |
| **Died in Care** | 21 | 0 | 63 | 13.76 | 13 | 4 | 4 |
| **Euthanized** | 6 | 4 | 731 | 170.67 | 1 | 0 | 5 |
| **2003** |  |  |  |  |  |  |  |
| **Released** | 31 | 0 | 439 | 58.35 | 9 | 9 | 13 |
| **Died in Care** | 50 | 0 | 573 | 26.18 | 29 | 16 | 5 |
| **Euthanized** | 4 | 4 | 77 | 44 | 1 | 0 | 3 |
| **2004** |  |  |  |  |  |  |  |
| **Released** | 27 | 0 | 200 | 55.78 | 9 | 2 | 16 |
| **Died in Care** | 39 | 0 | 2922 | 117.18 | 22 | 4 | 13 |
| **Euthanized** | 3 | 7 | 45 | 28 | 0 | 1 | 2 |
| **2005** |  |  |  |  |  |  |  |
| **Released** | 30 | 0 | 557 | 90.27 | 9 | 2 | 19 |
| **Died in Care** | 50 | 0 | 584 | 33.22 | 23 | 15 | 12 |
| **Euthanized** | 6 | 0 | 1440 | 246.33 | 3 | 2 | 1 |
| **2006** |  |  |  |  |  |  |  |
| **Released** | 41 | 0 | 538 | 57.39 | 15 | 6 | 20 |
| **Died in Care** | 83 | 0 | 1020 | 31.70 | 46 | 21 | 16 |
| **Euthanized** | 14 | 0 | 56 | 8.93 | 8 | 5 | 1 |
| **2007** |  |  |  |  |  |  |  |
| **Released** | 60 | 0 | 1098 | 125.63 | 9 | 6 | 45 |
| **Died in Care** | 130 | 0 | 292 | 16.47 | 75 | 35 | 20 |
| **Euthanized** | 32 | 0 | 1437 | 63.75 | 15 | 6 | 11 |
| **2008** |  |  |  |  |  |  |  |
| **Released** | 50 | 0 | 714 | 119.58 | 15 | 9 | 26 |
| **Died in Care** | 92 | 0 | 440 | 32.11 | 53 | 20 | 19 |
| **Euthanized** | 39 | 0 | 48 | 5.92 | 31 | 5 | 3 |
| **2009** |  |  |  |  |  |  |  |
| **Released** | 58 | 1 | 406 | 65.95 | 4 | 13 | 41 |
| **Died in Care** | 79 | 0 | 233 | 22.21 | 41 | 23 | 15 |
| **Euthanized** | 91 | 0 | 994 | 21.05 | 66 | 17 | 8 |
| **2010** |  |  |  |  |  |  |  |
| **Released** | 56 | 0 | 191 | 63.80 | 9 | 11 | 36 |
| **Died in Care** | 47 | 0 | 278 | 19.60 | 29 | 12 | 6 |
| **Euthanized** | 61 | 0 | 70 | 7.20 | 43 | 14 | 4 |
| **2011** |  |  |  |  |  |  |  |
| **Released** | 151 | 0 | 535 | 74.46 | 32 | 10 | 109 |
| **Died in Care** | 117 | 0 | 384 | 17.18 | 67 | 30 | 20 |
| **Euthanized** | 101 | 0 | 371 | 18.48 | 63 | 27 | 11 |
| **2012** |  |  |  |  |  |  |  |
| **Released** | 117 | 1 | 514 | 103.20 | 14 | 10 | 93 |
| **Died in Care** | 152 | 0 | 203 | 17.35 | 91 | 33 | 28 |
| **Euthanized** | 61 | 0 | 395 | 44.62 | 32 | 8 | 21 |
| **2013** |  |  |  |  |  |  |  |
| **Released** | 121 | 0 | 593 | 84.35 | 15 | 14 | 92 |
| **Died in Care** | 156 | 0 | 290 | 14.72 | 89 | 42 | 25 |
| **Euthanized** | 48 | 0 | 195 | 19.69 | 32 | 7 | 9 |
| **Total** |  |  |  |  |  |  |  |
| **Released** | 875 | 0 | 4919 | 105.3 | 165 | 106 | 604 |
| **Died in Care** | 1139 | 0 | 2922 | 30.89 | 634 | 2853 | 222 |
| **Euthanized** | 480 | 0 | 1140 | 31.51 | 298 | 98 | 84 |
