# Supplementary material for: Status of marine turtle rehabilitation in Queensland
Source: PeerJ. 2017 Mar 28;5:e3132. doi: 10.7717/peerj.3132 (PMC5372840; doi:10.7717/peerj.3132)
Supplement: Table S2 [file peerj-05-3132-s002.docx]

| **Cause of stranding** | **Died in Care** | **Euthanized** | **Released** |
| --- | --- | --- | --- |
| **Boat Strike/Fractures** | **49** | **60** | **37** |
| 0-7 | 29 | 45 | 5 |
| 7-28 | 9 | 4 | 4 |
| >28 | 11 | 11 | 28 |
| **Depredation** | **7** | **1** | **7** |
| 0-7 | 3 | 1 | 4 |
| 7-28 | 3 | 0 | 1 |
| >28 | 1 | 0 | 2 |
| **Disease** | **263** | **149** | **49** |
| 0-7 | 137 | 94 | 12 |
| 7-28 | 68 | 27 | 2 |
| >28 | 58 | 28 | 35 |
| **Dredging** | **1** | **0** | **0** |
| 0-7 | 1 | 0 | 0 |
| 7-28 | 0 | 0 | 0 |
| >28 | 0 | 0 | 0 |
| **Entangled Ghost fishing** | **0** | **1** | **0** |
| 0-7 | 0 | 1 | 0 |
| 7-28 | 0 | 0 | 0 |
| >28 | 0 | 0 | 0 |
| **Entanglement Crabbing** | **2** | **2** | **16** |
| 0-7 | 1 | 2 | 3 |
| 7-28 | 1 | 0 | 1 |
| >28 | 0 | 0 | 12 |
| **Entanglement fishing** | **16** | **12** | **30** |
| 0-7 | 8 | 10 | 5 |
| 7-28 | 5 | 1 | 7 |
| >28 | 3 | 1 | 18 |
| **Entanglement rope** | **5** | **2** | **4** |
| 0-7 | 5 | 1 | 0 |
| 7-28 | 0 | 1 | 1 |
| >28 | 0 | 0 | 3 |
| **Ingestion of foreign material** | **35** | **18** | **1** |
| 0-7 | 15 | 10 | 1 |
| 7-28 | 16 | 5 | 0 |
| >28 | 4 | 3 | 0 |
| **Netting** | **0** | **1** | **3** |
| 0-7 | 0 | 1 | 2 |
| 7-28 | 0 | 0 | 0 |
| >28 | 0 | 0 | 1 |
| **Other Anthropogenic** | **9** | **2** | **1** |
| 0-7 | 1 | 2 | 0 |
| 7-28 | 3 | 0 | 0 |
| >28 | 5 | 0 | 1 |
| **Shark Control Program** | **4** | **1** | **6** |
| 0-7 | 2 | 0 | 2 |
| 7-28 | 2 | 0 | 0 |
| >28 | 0 | 1 | 4 |
| **Unknown** | **744** | **231** | **361** |
| 0-7 | 430 | 131 | 93 |
| 7-28 | 174 | 60 | 50 |
| >28 | 140 | 40 | 218 |
| **Unknown Natural** | **4** | **0** | **2** |
| 0-7 | 2 | 0 | 2 |
| 7-28 | 2 | 0 | 0 |
| >28 | 0 | 0 | 0 |
| **Buoyancy Disorder** | **0** | **0** | **358** |
| 0-7 | 0 | 0 | 36 |
| 7-28 | 0 | 0 | 40 |
| >28 |  |  | 282 |
| **Grand Total** | **1139** | **480** | **875** |
